# Physical Forces Shape Group Identity of Swimming *Pseudomonas putida* Cells

**DOI:** 10.3389/fmicb.2016.01437

**Published:** 2016-09-16

**Authors:** David R. Espeso, Esteban Martínez-García, Víctor de Lorenzo, Ángel Goñi-Moreno

**Affiliations:** Systems Biology Program, Centro Nacional de Biotecnología – Consejo Superior de Investigaciones CientíficasMadrid, Spain

**Keywords:** bacterial swimming, macroscopic patterns, compression wave, media constraints, mechanical stress, physics simulation, *Pseudomonas putida*

## Abstract

The often striking macroscopic patterns developed by motile bacterial populations on agar plates are a consequence of the environmental conditions where the cells grow and spread. Parameters such as medium stiffness and nutrient concentration have been reported to alter cell swimming behavior, while mutual interactions among populations shape collective patterns. One commonly observed occurrence is the mutual inhibition of clonal bacteria when moving toward each other, which results in a distinct halt at a finite distance on the agar matrix before having direct contact. The dynamics behind this phenomenon (i.e., intolerance to mix in time and space with otherwise identical others) has been traditionally explained in terms of cell-to-cell competition/cooperation regarding nutrient availability. In this work, the same scenario has been revisited from an alternative perspective: the effect of the physical mechanics that frame the process, in particular the consequences of collisions between moving bacteria and the semi-solid matrix of the swimming medium. To this end, we set up a simple experimental system in which the swimming patterns of *Pseudomonas putida* were tested with different geometries and agar concentrations. A computational analysis framework that highlights cell-to-medium interactions was developed to fit experimental observations. Simulated outputs suggested that the medium is compressed in the direction of the bacterial front motion. This phenomenon generates what was termed a *compression wave* that goes through the medium preceding the swimming population and that determines the visible high-level pattern. Taken together, the data suggested that the mechanical effects of the bacteria moving through the medium created a factual barrier that impedes to merge with neighboring cells swimming from a different site. The resulting divide between otherwise clonal bacteria is thus brought about by physical forces—not genetic or metabolic programs.

## Introduction

Bacterial populations growing on surfaces tend to expand, according to environmental/experimental conditions, shaping different high-level patterns as, for instance, surface colonies, ramifications (Czirók et al., 1996), fractals (Fujikawa and Matsushita, 1989), or radial expansion (Bubendorfer et al., 2014). The mechanistic features of the environment where bacteria thrive are essential to determine the dynamics that rule the expansion through the physical space. In the case of liquid or low viscous media, bacteria use flagella to propel themselves, in a non-social manner, thereby exploring the surroundings for food in a movement. Such swimming has been traditionally studied to determine how the mechanics of the flagellar propulsion in liquid medium determines the macroscopic displacement (Macnab and Aizawa, 1984; Hyon et al., 2012) or in studies centered on exploring bacterial chemotactic behavior (Adler, 1966; Koster et al., 2012). Recent reports have expanded the area of research to the possible influence of the environmental conditions in such phenomena (Croze et al., 2011; Patteson et al., 2015).

Understanding bacterial spreading dynamics in soft materials is essential to solve problems associated with the presence of microorganisms in undesired contexts. Food spoilage (Sokunrotanak et al., 2013) or bacterial infections developed in immunocompromised patient’s tissues (Ki and Coleman, 2008; Sen et al., 2009) are two examples where the lack of knowledge in this area contributes to increase economic loses and serious health problems.

Structuring macroscopic patterns by bacteria can be driven by several factors. Cell–cell interactions are among the most decisive actors behind pattern formation (Strassmann et al., 2011). The cooperation of relatives in some bacterial strains, through the so-called kin-discrimination systems (Lyons et al., 2016), seem lead to diversification within species which, in turn, is a major cause of different multicellular behaviors. The origins of such kinship is often explained by only just chemical causes, as quorum-sensing signals (Schluter et al., 2016). However, these studies leave out the effects produced by other physical phenomena involved in cell–cell and/or cell–medium interactions despite the fact there are strong evidences in literature—pointing that this approach underestimates their influence. Numerous theoretical studies explore group formation and density distribution of bacterial populations in liquids studying parameters such as orientation and spatial restriction (Dombrowski et al., 2004; Zhang et al., 2010; Marchetti et al., 2013). Works studying swimming of bacterial colonies through microporous materials already reported a bacterial dynamics dependent of the hydrodynamics and surface properties (Berg and Turner, 1990; Hornberger et al., 1992; Hu et al., 2015). Experiments describing biofilm formation in wet channels (van Loosdrecht et al., 2002; Rusconi et al., 2011; Espeso et al., 2016) or soft surfaces (Ben-Jacob et al., 1994; Asally et al., 2012) highlight the importance of cell-to-environment interactions in bacterial spreading dynamics. Furthermore, studies in tissue morphogenesis and swimming (Campàs et al., 2014; Patteson et al., 2015) have proved that mechanical forces play a critical role in pattern organization and bacterial displacement.

In the course of the experiments with the soil bacterium *Pseudomonas putida* KT2440 (Bagdasarian et al., 1981), we observed that two swimming populations in a soft agar medium halt at a finite distance before engaging in contact; therefore, they avoid mixing at macroscopic level, resembling the so-called Dienes line (Dienes, 1946, 1947), initially noted in swarming populations of different strains of *Proteus mirabilis*. The use of clonal populations here suggested that kin discrimination systems cannot play a role in shaping the observed patterns. Nonetheless, the ability to distinguish self from non-self recalled an identity-like behavior (Lyon, 2015) that was worth analyzing further. Similar effects on different bacterial species have previously been mathematically modeled as a consequence of insufficient nutrient availability (Sekowska et al., 2009). However, no evidence was found to correlate the halting behavior with nutrient availability in *P. putida* KT2440.

As an alternative, the analysis presented below focuses on pure mechanical interactions; not only among cells but also between cells and medium (Duffy and Ford, 1997). It has been reported that swimming cells at a certain speed have a specific *cost of moving* (Mitchell and Kogure, 2006) which is, in turn, influenced by the viscosity/rigidity of the medium. Furthermore, when swimming cells form dense populations, where the bacteria are close enough, they push against the medium generating flows in it (Wolgemuth, 2008). These two observations suggest that, depending on the state of the medium, the pushing forces would need to be stronger (higher moving cost) or weaker (lower moving cost) to effectively have an impact of the flows, thus leading to population expansion. Recent results showed that even weak swimmers have a not-insignificant effect on the fluid/medium (McDonnell et al., 2015) reinforcing the need to analyze the mechanical effects of cell–medium interactions on the observed patterns. In this context, the results below show that the mechanical effects of the bacteria moving through the medium suffice to explain the gap that impedes two or more swimming populations to merge at the site of confluence.

## Materials and Methods

### Bacterial Strains and Growth Conditions

The strains used in this study are fluorescently labeled derivatives of *P. putida* KT2440 (Bagdasarian et al., 1981). These strains were tagged by the used of the mini-Tn*7* system (Koch et al., 2001; Rochat et al., 2010) that insert the fluorescent marker at an intergenic and neutral site of the KT2440 genome and constitutively express either the green fluorescent protein (KT-GFP) or mCherry (KT-mCherry).

*Pseudomonas putida* cells were routinely grown on M9 minimal medium (Sambrook et al., 1989) supplemented with 0.2% (w/v) glucose as carbon source and incubated at 30°C with aeration. When required gentamicin was added at 10 μg ml^-1^ as final concentration. Phosphate-buffered saline (PBS; 8 mM Na_2_HPO_4_, 1.5 mM KH_2_PO_4_, 3 mM KCl, and 137 mM NaCl, pH 7) was used to dilute cells.

### Swimming Assays, Imaging, and Quantification

Unless otherwise indicated swimming plates were prepared using fresh M9 minimal media supplemented with 0.2% (w/v) glucose and 0.2% (w/v) casamino acids (CAA), and solidified with 0.3% (w/v) agar. Some experiments required adding different concentrations of glucose ranging from 0.05 to 0.5% (w/v), CAA from 0.05 to 0.2% (w/v), and agar from 0.15 to 0.45% (w/v). Customized geometry plates were performed with 32 and 90 mm diameter circular Petri dishes. The plates were first filled with M9 culture medium solidified with 1.0% (w/v) agar, then cut to create the customized shape gap, which was finally filled with M9 at a final concentration of 0.3% (w/v) agar.

For motility assays on soft agar plates, bacterial cells were grown overnight in 3 ml of M9 minimal medium supplemented with 0.2% (w/v) glucose at 30°C with shaking (170 rpm). Then, cultures diluted to an optical density (OD) of 0.1 in 1 ml of PBS and 2 μl spotted on the surface of the soft agar plate. To assay the influence of the cells in the swimming patterns the OD was adjusted properly (0.01, 1, 10, and 100). Swimming plates were prepared on the same day of the assay. We used either 90 mm diameter Petri dishes containing 20 ml of media or 35 mm × 10 mm polystyrene tissue culture dish (Falcon) filled with 3 ml of media. Plates were incubated at room temperature for 24 h, 48 h, and up to 6 days in closed plastic containers with wet tissue papers to maintain moisture and to avoid desiccation of media throughout the experiment. At indicated points, plates were photographed using a Nikon D3X with an AF Micro Nikkor 60 mm f/2.8D objective. To quantify the expansion of the colonies, pictures were first processed with Photoshop to extract the inner part of the plate and then imported to MATLAB (The MathWorks, Inc.). Thus, images were gray-scale converted, filtered, when needed, and binarized using manual thresholding. Then, the occupied zone of the colonies was calculated and normalized to the total area of the plate for each picture. Finally, the average and standard deviation plotted.

### Physics-Based Simulation Environment

In order to match observations at the macroscopic level with physical qualities of individual cells, the simulation strategy focused on discrete objects for both population(s) and medium to reproduce the motion of the bacteria through soft agar. Instead of having a single object to simulate an expanding (growing and swimming) colony, the simulation used several circular objects so that the overall structure could adapt its morphology to environmental constraints. Importantly, any given cellular-object did not represent a single cell, but a group of cells.

The simulation made use of the 2D rigid body physics library Chipmunk^1^ through its Python wrap Pymunk^2^. This physics engine takes care of all collisions among shapes happening in the model. High accuracy is needed in solving collisions as the simulation relies on the effects of these events to shape the patterns. The simulation was built upon the physics engine Chipmunk, that uses the Gilbert–Johnson–Keerthi algorithm (Gilbert et al., 1988) to calculate distances between objects and the Expanding Polytope Algorithm to calculate penetrations (Bergen and Bergen, 2003). Upon collisions, an impulse is generated on the objects (consequence of the mechanical stress) that pushes the objects apart (measured in **Figure 2C**) and added to the velocity of the object to finally calculate the new moving vector.

The algorithm developed to simulate the patterns is sketched as follows:


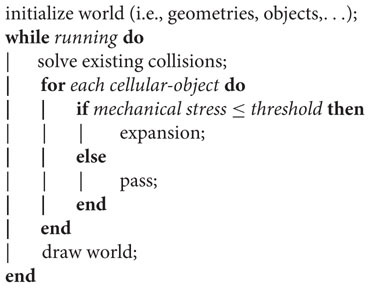


where, after initialization of the simulated world, the cellular-objects expand if, and only if, the mechanical stress on them is lower than a predefined threshold (measured in dimensionless pressure forces units). The simulation only enters a new for loop after a period of time (proportional to the number of cellular-objects present at that iteration), so that Chipmunk’s collision solver has time enough to resolve object overlaps and avoid simulation inconsistencies.

The expansion of a cellular-object is simulated by creating a new one on the same spatial coordinates; the size of the objects is constant. After each expansion event the two objects push each other apart, process which simulate both swimming and growing. Medium objects (agar units) did not play an active role in the algorithm and were just used by the collision solver to update positions.

## Results

### Pattern Formation in Swimming Populations of *P. putida*

**FIGURE 1:**
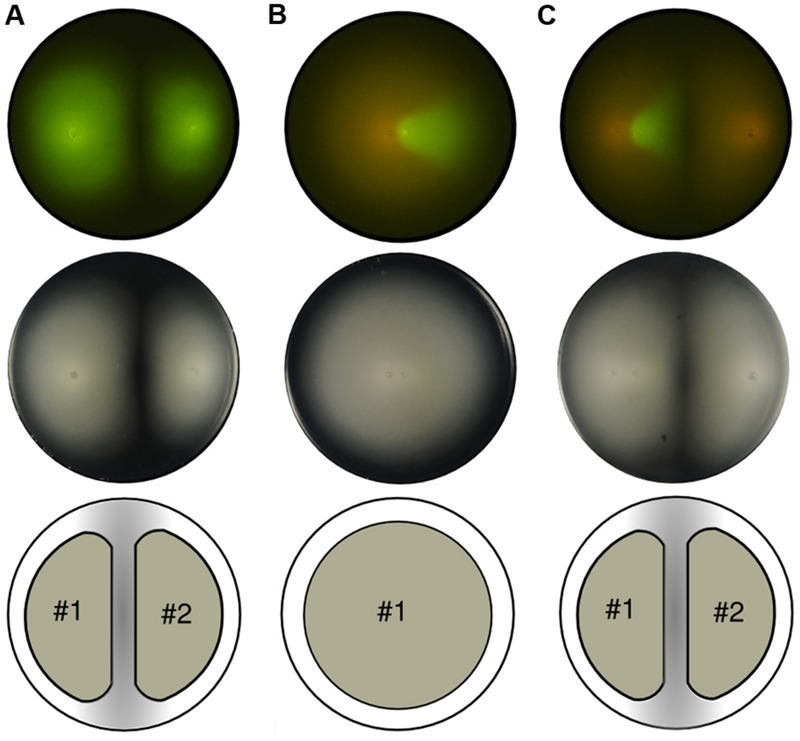
**Swimming patterns of *P. putida* KT2440 populations inoculated on soft agar plates.** Top row: fluorescent capture. Middle row: phase contrast image. Bottom row: unified expansion group. **(A)** Two clonal green fluorescent protein (GFP)-labeled colonies grow and swim maintaining the empty area in the confluence and establishing two distinct subgroups. **(B)** Two populations, GFP and mCherry-labeled, inoculated close to each other. While colonies do not mix they grow as a single group, thus expanding radially from the center. **(C)** Soft agar plate with three inocula (two close to each other while the third apart from them). The two populations that were inoculated closely (left part of the picture) reproduce the previous scenario shown in **(B)**, thus evolving as a single group, while the mCherry-labeled colony growing from the right part of the plate forms a second group.

Under specific medium conditions, which will be described later in this section, the bacterium *P. putida* swims freely within soft agar plates. The only limit to their, otherwise radially, expansion is formed by physical obstacles such as the plastic plate wall and, surprisingly, the presence of other expanding colonies. While the former obstacle is intuitively obvious, the latter deserves more attention because its causes are not straightforward. First, to rule out multi-strain differentiation or non-kin detection systems as the possible cause the same strain was inoculated twice on an agar plate at *long* distance (relative to the plate diameter; **Figure 1A**). It was observed that both populations swam to fill the space available until reaching a halting point in which an apparently empty stripe in the middle of the plate was clearly drawn and visible to the naked eye. The pattern was perfectly shaped and two groups were visually identified in the agar plate. The fact that the cells are genetically identical at both sides of the stripe indicated the mechanism behind their behavior is not due to, for instance, different extracellular signaling molecules secreted to the environment. Indeed, it seems inappropriate for a cell to *decide* to use one signaling system or the other depending on the spatial position of its inoculum (left or right).

However, when the cells were inoculated in close proximity (**Figure 1B**), the unoccupied space between swimming colonies did not emerge and the cells swam as one single group rather than two separate ones. For visualization purposes, the second inoculum had a version of the GFP-labeled strain with mCherry instead (see Materials and Methods). In essence, both strains are still genetically equal. In consideration of the above, the lack of separation between populations must be related to the only parameter changed in this experiment: the distance between initial inocula. This pointed out that the mechanism responsible for the separation depends on the growing state of the colonies and, most importantly, on their swimming activity record.

Visualization of the distance-dependent effect on an agar plate with three inocula confirmed previous suggestions (**Figure 1C**). With two populations swimming from close initial spots, and one from a distant location, two single groups were finally observed, as in the first experiment. The first group is formed by two strains (GFP and mCherry-labeled), that in the phase contrast image looks indistinguishable from the shape developed by one unique strain. The second group, formed by one single strain (mCherry-labeled), starts its expansion from far enough distance to grow as an independent population; therefore, it halted before getting in contact with the former consortia. Being that halting distance so large (in comparison with a cell’s length) suggests that the bacteria of each group do not get in touch at a macroscopic level, and the mechanisms behind these patterns are not likely to be exclusively based on cell–cell interactions.

### Computational Inspection of the Cell–Medium Interplay

Going beyond cell–cell interactions implied that the dynamic role played by the medium on the cells or, more specifically, on their swimming behavior needed to be consider. For that purpose, a simulation environment (full details in Section “Materials and Methods”) was developed based on physical interactions (i.e., pushing forces) between two types of objects, representing (groups of-) cells and medium units. Both types of objects are circular shapes that were initially situated on a two-dimensional map following an ordered distribution and specific behavioral rules. On the one hand, cellular objects, that do not represent single cells, were grouped together around inoculum coordinates to emulate the experimental starting point. This *cellular*-type is able to expand (similar to cell division) under a specific condition related to its mechanical stress, also referred to as *pressure* in this text: if, and only if, the object is subjected to lower pressure (understood as the physical force being applied to its surface), than a predefined threshold. Otherwise the object is considered non-active, representing a cellular group that does not expand further. Importantly, the expansion of a cellular-object is a discrete computational strategy to simulate bacterial spatial spreading due to both cellular division and flagella-driven radial displacement observed experimentally. On the other hand, medium objects were initially distributed uniformly across the space and do not present any kind of active behavior (i.e., expansion).

**FIGURE 2:**
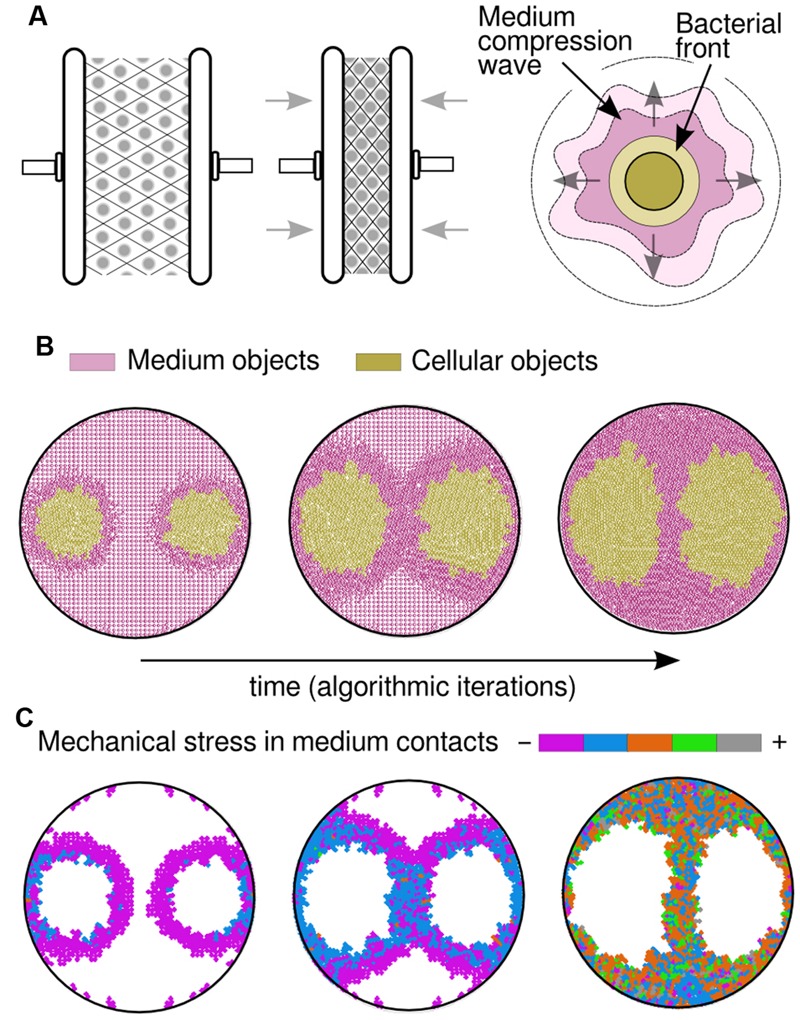
**Simulation results by considering medium physical constraints. (A)** By incorporating compressible medium dynamics to our simulations, so that it gets condensed when squeezed (left and middle drawings), the correct patterns arose. As the bacterial front moves it produces what was termed a compression wave (right drawing), a thicker layer in the medium that plays its own role in pattern formation. **(B)** Visualization of a compression wave in a simulation (time increases from left to right) where both medium and cells are represented by circular objects. The medium (colored in pink) is compressed as the bacterial populations (colored in gold) apply pressure on it due to pushing forces; meanwhile, populations tend to spread toward low-compressed areas of the plate, leading to the experimentally observed patterns. **(C)** Mechanical stress that is measured in medium objects, by adding the value of all pressure forces applied to any single object. At the beginning of the simulation (left) the majority of the objects remain still and only those in close proximity to the growing colony are being slightly compressed (purple). In a middle stage, the pressure increases in the bacteria that are close to the walls and those in between colonies. Finally (right), the colony halts when the medium is already highly compressed.

At the very beginning of a simulation, all circular objects (cells and medium) remain still. However, cellular-type objects will eventually expand resulting in an increase of the region covered by them. On that moment, the newly created cellular-type object(s) accommodate themselves in the two-dimensional surface; process in which they would need to push already existing objects apart. In turn, those objects being moved may as well push other ones (either cells or medium), and so on. Finally, a number of moving events will take place until all forces in the simulated space void each other and all objects, again, remain still. This approach implies that not only the cells are involved in shaping the final pattern but also the medium by applying weaker or stronger resistance to pushing forces. Simulated objects are not malleable (they cannot be deformed) but the global structure is—due to the gaps between shapes. This phenomenon (**Figure 2A**) provides the medium structure with a decisive characteristic to the simulation, termed *compressibility*, which is a measure for volume variation observed in the medium when is compressed. Importantly, the limit for the compression is defined by the amount of empty space; once the gaps disappear, the structure cannot be compressed further. This simple computational framework is surprisingly close to experimental reality: the compressibility is equivalent to the inverse of the bulk modulus of a material (measuring the natural resistance of a material against hydrostatic pressure). That means the model is implicitly taking into account this mechanical property for the medium. Moreover, several studies (Hjertén, 1961; Righetti et al., 1981; Johnson and Deen, 1996; Le Goff et al., 2015) reported agar as a porous solid matrix filled with liquid showing complex viscoelastic properties, features being captured by the interspace between medium objects, its resistance to be moved and the expansion threshold defined above.

### Medium Deformation Due to Pushing Forces and Compressible Dynamics

It is of common usage, specially within the swimming/swarming literature, the term *bacterial front* to make reference to the cells that, being at the edge of a population, move forward into cell-empty space. The simulation suggests that this front has a deforming effect in the structure of the medium layer that is situated immediately next to it and ahead of its direction. It also suggests that this effect is passed on to the next medium layer, and so on, in what we have termed a *compression wave* (**Figure 2A**). Such scenario allowed, for the first time, to computationally reproduce the patterns obtained in swim experiments without considering any cell–cell biochemical interaction. Under this frame, the cells at both sides of the separation stripe do not need to be in physical contact to sense each other, which is a key feature of the problem. Instead, medium compression would somehow hinder advance of cells either because of a mere physical constrain or because of stress sensor(s) that inhibit bacterial motility. This hypothesis suggests a general mechanism for bacteria swimming in enclosed spaces: physical and mechanical constraints may not only govern the dynamics of the experimental design described in this work with *P. putida* KT2440, but other microbial species as well. Along the line, swimming experiments performed with bacteria such as *Pseudomonas aeruginosa* PAO1 (Stover et al., 2000) and *E. coli* MG1655 (Blattner et al., 1997), faithfully reproduced the separation pattern observed with *P. putida* KT2440 (see Supplementary Figure S1), in a fashion consistent with the hereby proposed hypotheses. Results of a simulation over time are captured in **Figure 2B**, where two populations were inoculated at distant locations (similar to the experiment in **Figure 1A**) within a soft agar plate. At the beginning, the populations swim radially while they push apart medium objects in the same direction. As a result, the medium started being more compressed in those areas surrounding the colony (darker region in figures). At a middle stage through the simulation process, the medium was compressed to the limit in specific areas and that had a decisive role in shaping multicellular patterns. Indeed, the medium between the two colonies cannot be compressed further so the cells found less mechanical resistance in swimming toward the top and bottom of the plate. Finally, the entirety of the surface was highly compressed and the cells reached the halting point, returning output patterns that are similar to experimental observations.

**FIGURE 3:**
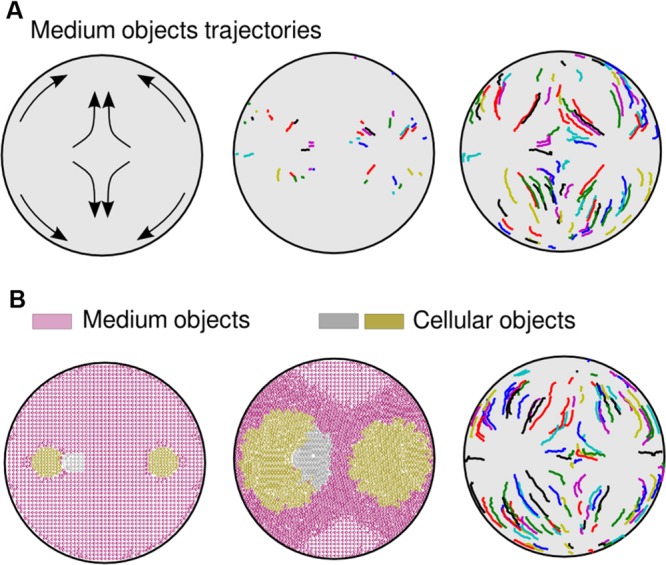
**Medium trajectories within an agar plate. (A)** Trajectories in a simulation where two bacterial populations where inoculated. Initial predictions (left part of the picture) were validated through computational analysis. During the first steps of the simulation (middle), with no obstacles, medium objects move radially. When obstacles enter into the process (right), such as waves traveling in opposite direction or walls, medium units change the moving routes accordingly, going toward less compressed areas (bottom and top part of the plate). **(B)** Simulation that resembles the experiment shown in **Figure 1C**. Although three populations were inoculated (left part of the picture), the cells only formed two separate groups (center). Trajectories of medium objects (right) do not differ substantially from the previous two-population simulation **(A)**.

The accumulation in time of mechanical stresses applied to objects (**Figure 2C**) shows those areas of the medium that, while surrounding the swimming cells, are less compressed; not surprisingly situated at the top/bottom of each population. Compression started close to the Petri dish walls (in **Figure 2C**, first orange objects appearance) and spread along its circumference, following the same trajectories as medium objects (**Figure 3A**). As the simulation progressed, the two opposing compression waves joined in between populations and the local forces increased in that region, leaving only two directions (up/down) with lower mechanical pressure. Apart from the middle stripe, the topology of the plate facilitated the triangle shape of the motionless areas (up/down), also noticeable in experimental observations. Finally, the impulse on all medium objects was high enough across the plate to result in a halting point for the cells, which stopped swimming. A simulation with three initial populations (**Figure 3B**) that resembled the experiment of **Figure 1C**, confirmed that two inocula close to each other swim as a single group in all respects, also in the way the cells move the medium. That move was produced radially at first and followed an up/down trajectory upon collisions with either plate walls or a confronting deformation wave.

### Effects of Initial Conditions on Swimming Patterns

**FIGURE 4:**
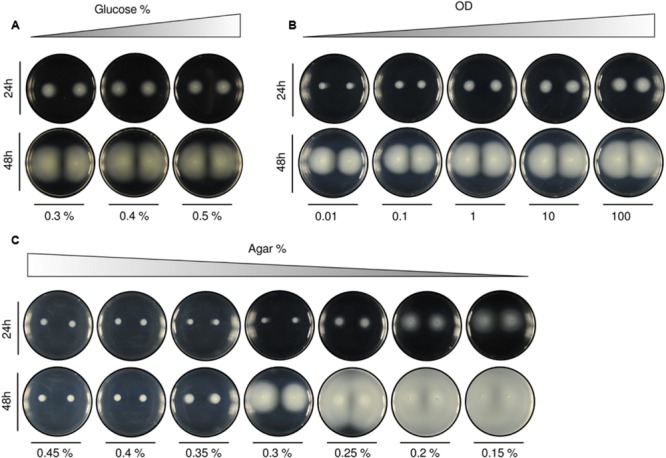
**Alteration of swimming patterns according to different initial setups. (A)** Variation in the quantity of carbon source [with agar at 0.3% (w/v) and OD at 0.1]. Increasing the percentage of glucose produced only small differences in the area covered by the colonies. **(B)** Tenfold increments in the optical density of initial inocula [with agar at 0.3% (w/v) and glucose at 0.2% (w/v)] resulted in bigger patterns. Population size across samples looks gradually proportional. **(C)** Agar variation [with glucose at 0.2% (w/v) and OD at 0.1]. The increase in the percentage of agar in the medium revealed itself as the most influential parameter for swimming pattern formation. We found two important thresholds: (1) above 0.3% (w/v) the medium was mostly solid and no swimming behaviors were observed for the tested experimental time, and (2) below 0.2% (w/v) the medium was essentially liquid and the patterns were hardly reproduced, resulting in a plate fully covered by cells. A quantification of the swimming normalized area of the colonies for the different experimental conditions **(A–C)**, performed as indicated in Section “Materials and Methods,” is included in Supplementary Figure S2.

Three parameters are mainly responsible for the swimming behavior: (i) nutrients (glucose), (ii) number of cells in the initial inoculum (OD), and (iii) medium stiffness (agar concentration). Previous experiments were prepared with glucose at 0.2% (w/v), 2 μl inoculum of a cell suspension at an OD of 0.1, and agar at 0.3% (w/v; more details on plate preparation in Section “Materials and Methods”) and **Figure 4** shows gradual variations in each of these parameters. Increase in the amount of glucose (**Figure 4A**) produced a mild effect in swimming expansion and exhibited a large variability between samples (specially at higher glucose concentrations; Supplementary Figure S2). This behavior was interpreted by the simulation framework (see below) as the consequence of a mere force balance between cells and medium objects. On a separate set of experiments, 10-fold increments in the number of cells in initial inocula (OD, **Figure 4B**) produced increasingly bigger patterns in linear proportion.

Although nutrients availability and OD are important parameters, it was agar concentration that was revealed as the most limiting factor. The studied patterns were only reproduced within a very specific agar % range (**Figure 4C**). At high percentages [>0.3% (w/v)] the medium became rigid and mostly solid. Under this scenario, the cells could not swim. On the contrary, at low agar concentrations [<0.25% (w/v)] the medium turned essentially liquid and the cells spread fast through all the plate.

### Mechanical Features of Simulated Cell–Medium Interactions

Once the swimming behavior of *P. putida* had been described in detail, a few subtle questions arose. What is the threshold that separates a liquid or a solid medium and its influence on the patterns? In an attempt to answer this question, changes in three different parameters of the simulation were done, namely: total number of medium objects, expansion thresholds and mass of medium objects. The model could qualitatively simulate and predict experimental results by just modulating these three computational parameters. The number of objects measured the medium degree of mobility, that is, the ease of relocating a unit of medium mass across the space. The mass of the object set the resistance for such medium unit to be displaced inside the computational simulation space. And the expansion threshold established what is the maximum mechanical stress above which the medium will not allow further expansion of cellular objects. The computational analysis suggested that the appearance of patterns and their shape are the direct consequence of a competition among all physical forces at stake and their resolution.

**FIGURE 5:**
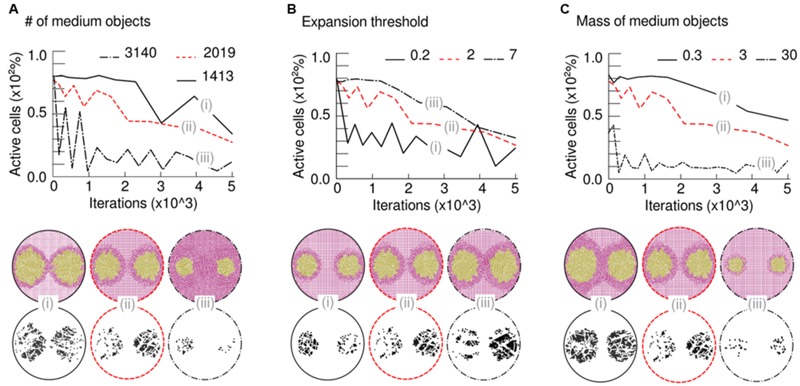
**Mechanical properties responsible for simulated patterns.** Top part: time-course lines represent number of active cells (those ones expanding) over time (iterations of the algorithm) in three runs with different conditions. Bottom part: simulation screenshots at the last iteration and detail of remaining active cells. **(A)** Variation in medium density is represented by alteration in the number of simulated medium bodies [*n* = 1413, (i); *n* = 2019, (ii); and *n* = 3140, (iii)]. As the density of the medium increases, the number of active cells decreases due to higher impulses (pressure) on them; therefore, the patterns do not arise. **(B)** The ease with which the colony modifies the medium is given by a pressure-dependent threshold that allows (or not) each cell to growth. When the threshold increases [*n* = 0.2, (i); *n* = 2, (ii); and *n* = 7, (iii)], cells resist environmental pressure better and the colony is *stronger*, thus able to grow faster and further. **(C)** Changes in the mass of medium bodies, leaving the total number fixed. As expected, an increase in this parameter [*n* = 0.3, (i); *n* = 3, (ii); and *n* = 30, (iii)] led to non-swimming colonies due to excessive pressure from the medium on growing cells.

Firstly, **Figure 5A** shows the effects of changes in medium density (**Figure 5A**). As medium objects were homogeneously distributed across the plate, an increase in their total number recreated a highly compressed medium with less empty space in between objects that would correspond to a more complex agar network (more tortuous, less porous, and more rigid to displacements). At a given time after the beginning of the simulation the sample with less medium objects [*n* = 1413, **Figure 5A** (i); and *n* = 2019, **Figure 5A** (ii)] had expanded further, and count on more active cellular-objects, than the sample in which the medium was already close to its compressibility limit from the start [*n* = 3140 objects, **Figure 5A** (iii)]. **Figure 5B** analyze the effects derived from alterations in the expansion threshold. The value of this threshold is programmed in each cellular-object allowing to expand (double) if, and only if, the mechanical stress on the object at a given time is below that number. In this case, the medium density is the same in all samples so that its pressure on the cells is directly comparable. As expected, a larger threshold [*n* = 7, **Figure 5B** (iii)] produced a more compressible medium; therefore, the produced patterns are bigger at the same time. On the contrary, small threshold values [*n* = 0.2, **Figure 5B** (i); and *n* = 2, **Figure 5B** (ii)] hardly allowed the cellular mass to expand because medium rapidly constrains the mobility of the swimming colony. Finally, **Figure 5C** explains what would happen as the mass of medium objects increase. After the same number of iterations, the sample with lighter objects [*n* = 0.3 dimensionless mass, **Figure 5C** (i)] showed bigger macroscopic patterns than the simulation with heavier ones [*n* = 3, **Figure 5C** (ii); and *n* = 30, **Figure 5C** (iii)]. The heavier the medium-object was, the more resistance it presented to be moved and, therefore, a cellular-object that tries to push it reached its threshold earlier.

Simulation results point out possible mechanisms to control pattern formation and their correlation. For instance, medium density (**Figure 5A**) and mass of medium objects (**Figure 5C**) are similar parameters, in terms of their effects, as an increase in them will produce inversely proportional macroscopic patterns. On the contrary, the maximum stress supported by the agar medium before its compression limit (threshold, **Figure 5B**) is directly proportional to the patterns’ expansion. Caution is recommended in theorizing the *in vivo* significance of previous parameters, as there are still open questions to be solved. Establishing a direct relationship between experimental and simulated setups is not straightforward, as agar structural and elastic properties are mutually dependent (modifications in the structure would have an impact in the elastic properties, and *vice versa*). The model successfully captured the qualitative mechanics behind the observed patterns. However, quantification is out of the scope of the present model, as the nature of the physical variables acting on the medium is largely unknown. All in all, the analysis suggested all patterns observed experimentally are the consequence of the balance, within the population, of pure mechanical forces.

### Role of Mechanical Stresses in Swimming Patterns: Influence of Expansion Area Geometries and Bacterial Density

Under the frame proposed above, compression waves formed in front of bacterial colonies would be responsible for medium reallocation and increase of mechanical stress that stops bacterial spreading when two such waves collide. A deeper look at the composition of soft agar medium (a porous elastic solid matrix filled with liquid) raised questions on the target of that mechanical stress. Studies performed with other hydrogels, such as collagen or polyacrylamide, in the context of cell–medium interaction (Vaughan et al., 2013; Denisin and Pruitt, 2016) suggest that swelling and shrinking phenomena observed in hydrogels can be explained as a fluid–solid mechanical interaction in which stresses applied to the solid induces liquid displacement inside the gel depending on its microstructural and elastic properties. And reported measurements of pore size for agarose hydrogels using different techniques are discordant. Pore size diameter values around 500 nm are given in Righetti et al. (1981) for agarose concentration as small as 0.15% (w/v), but Narayanan et al. (2006) reported a pore size diameter of 600 nm for agarose concentrations around 1% (w/v). This scenario points that medium displacement induced by the expanding colonies can affect either the solid or the liquid fraction in the hydrogel.

Although the physical details responsible for medium compression were not described at a micro-scale, their effects at a macro-scale level were precisely predicted and tested. A set of experiments where the geometry of the bacterial expansion niche and the bacterial concentration was modified, confirmed the relationship between observed patterns and specific stress configurations.

**FIGURE 6:**
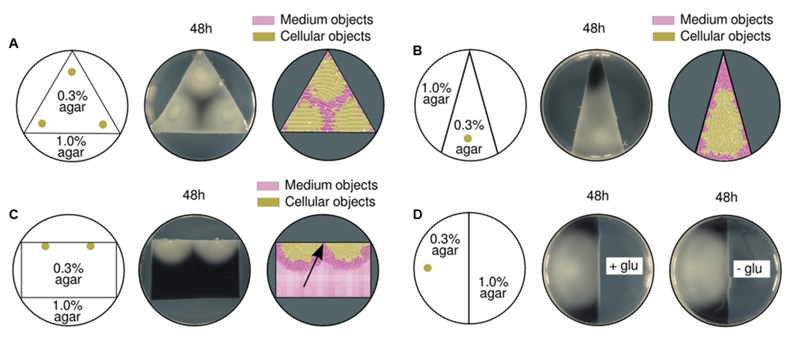
**Effects of different swimming area geometries in pattern formation. (A)** A triangular region with swimming conditions [agar at 0.3% (w/v)] was surrounded by *hard* medium [agar at 1.0% (w/v)] impossible to swim through. Swimming results of three initial inocula, close to each triangle vertex (left), are shown in experimental (middle), and simulation (right) setups. The empty area in the middle of the three inocula is a direct consequence of the physical environmental constraints (i.e., angles) where the populations grew. **(B)** Triangular region with an acute angle on top; a single population initiated its expansion from the bottom (left). In both experimental (middle) and simulated (right) scenarios, the medium is compressed toward the upper area of the triangle. **(C)** Two populations were inoculated at the edge of a rectangular swimming area (left). Experimental (middle) and computational (right) results show that the colonies did actually get in contact (pointed by a black arrow). In simulations, the medium in between populations was washed out toward the bottom of the rectangle. **(D)** A single population swam from soft to hard medium [agar at 0.3% (w/v) and 1.0% (w/v), respectively; left] in two replicas with and without glucose (middle and right). There were no differences in the behavior of cells when approaching hard agar regions, which suggested that a glucose-free environment does not have an influence in stripe formation.

Specific boundaries for the swimming region were imposed within a single Petri dish plate by customizing its geometry (see Materials and Methods). The triangular shape showed in **Figure 6A** allowed to confirm, firstly, the ability to confine a swimming population within a predefined area by just altering the agar concentration of the medium. That is to say, fluctuations in medium compressibility across the surface will fundamentally influence the expansion range of a population. Secondly, the experiment showed that the empty space in between populations (three inocula close to vertices) was precisely predicted by simulations; the arising patterns were a direct consequence of both the topology of the space and the coordinates of the inocula. Having in mind that rather simple mechanistic behavior, it was intuitively predicted that a single inoculum close to the shortest edge of an isosceles triangle would compress the medium toward the acute angle. Simulated and experimental results confirm the latter prediction (**Figure 6B**), indicating that initial symmetric setups (in both geometries and inocula coordinates) will consequently produce symmetric patterns.

In order to modify the initial radial expansion, two populations were inoculated at the edge of a rectangular swimming area to promote semi-circular growth (**Figure 6C**). As predicted, the two populations generated such a semicircular front pushing the medium crosswise rather than directly toward each other. As a consequence, the medium objects (as measured in the simulated scenario, right part of the figure) did not accumulate between colonies but moved toward the bottom of the plate. The populations did actually meet at the middle point instead of reaching the halting distance like originally observed in **Figure 1**.

On an attempt to analyze the role played by potential nutrient gradients, a single population was inoculated on a semicircular swimming area while the rest of the plate was nutrient-rich or nutrient-free in two different samples (**Figure 6D**). In both cases, the population swam to the limit and did not stop at a finite distance before the nutrient-free region. This result suggests the cells were not sensitive to nutrient decrease before reaching that frontier; therefore, nutrient-oriented explanations are unlikely to fit observations.

## Discussion

Understanding multicellular behaviors will ultimately benefit many fields within the realm of Microbiology. To this end, one needs to clearly distinguish which phenomena stem from a genuine biological program or adaptive behavior and which are the result of mere physical forces at works in the scenario under study. Pattern formation on plates thus needs to be addressed from an interdisciplinary point of view. Many recent efforts focus their study on describing biochemical solutions (Sekowska et al., 2009; Strassmann et al., 2011; Lyons et al., 2016; Schluter et al., 2016), overrating the effects of physical causes (Duffy and Ford, 1997; Mitchell and Kogure, 2006; Wolgemuth, 2008; McDonnell et al., 2015).

Swimming populations of *P. putida* KT2440 expanding toward each other halt at a finite distance before both bacterial fronts become into contact. Consequently, a cell-free boundary is formed within the two populations several orders of magnitude wider than a bacterial cell. Inter-cellular communication among fronts or cell differentiation seems improbable as a possible cause of such growth exclusion at the border (all cells are clonal). The work above explores instead mechanical features of cell–cell and cell–medium interactions. To this end, simulation software, based on a physics engine (Chipmunk) for collision handling, was developed where the medium is compressible, thus deformable to a limit. Simulation analysis suggested the cells (swimming as a highly crowded block) were able to induce medium displacement to the extent of generating compression fluctuations in different medium locations. In turn, these differences in compression interact with the cells as follows: the less compressed a region is, the more likely the cells will swim through it. As a result, the experimentally observed patterns were computationally reproduced with great detail.

In sum, both experimental and theoretical data presented above support the notion of a physics-only explanation of the macroscopic observations at stake. In this context, we advocate consideration of physical features of cell–medium interactions—not only biological aspects, in the analysis of multicellular behaviors. Along the same line, the ability of an expanding, mobile population to create a factual exclusion barrier around it may expose a thus far unnoticed angle of bacterial sociology. In particular, the mechanical effects of motion through a semisolid medium seems to contribute to emergence of a neighborhood-based group identity that curbs territorial invasion by both kin and non-kin counterparts. We thus believe that the work above provides new elements for modeling complex social scenarios with *á la carte* bacterial players.

## Author Contributions

DR-E and EM-G developed the concept, made experiments, and contributed to paper writing. AG-M masterminded the work, set up the computational frame, and contributed to paper writing. VL directed the project and contributed to paper writing.

## Conflict of Interest Statement

The authors declare that the research was conducted in the absence of any commercial or financial relationships that could be construed as a potential conflict of interest.

## Footnotes

1 http://chipmunk-physics.net/

2 http://www.pymunk.org
