# Supplementary material for: Physical Forces Shape Group Identity of Swimming Pseudomonas putida Cells
Source: Front Microbiol. 2016 Sep 16;7:1437. doi: 10.3389/fmicb.2016.01437 (PMC5025637; doi:10.3389/fmicb.2016.01437)
Supplement: Supplementary file 1 [file Image_1.PDF]

Supplementary Fig. S1 to Espeso *et al.* (*Physical forces shape group identity of swimming Pseudomonas putida cells*)

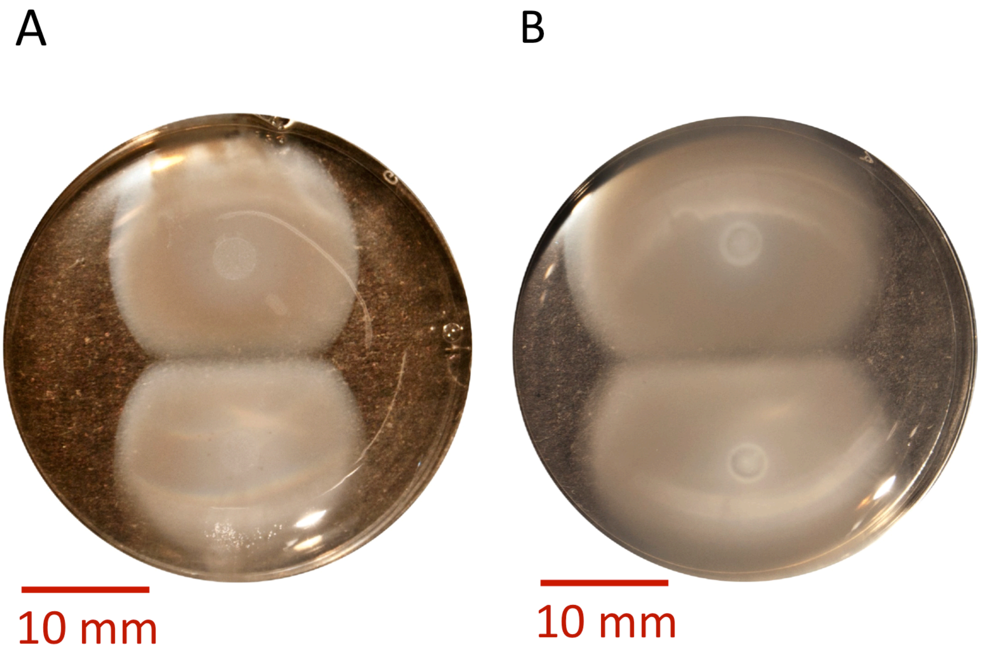

Swimming patterns formed by bacteria other than *Pseudomonas putida* KT2440. Swimming experiments performed using *E.coli* MG1655 (A) or *P. aeruginosa* PAO1 (B). Swimming plates were prepared with M9 minimal media solidified with agar at 0.3% (w/v) and supplemented with 0.2% (w/v) glucose and 0.2% (w/v) casamino acids. Both samples were inoculated using 2 µl of an overnight cell suspension adjusted to an OD_{600} of 0.1. Images were taken after 48 h and the contrast enhanced for a better visualization.
