# Supplementary material for: Physical Forces Shape Group Identity of Swimming Pseudomonas putida Cells
Source: Front Microbiol. 2016 Sep 16;7:1437. doi: 10.3389/fmicb.2016.01437 (PMC5025637; doi:10.3389/fmicb.2016.01437)
Supplement: Supplementary file 2 [file Image_2.PDF]

Supplementary Fig. S2 to Espeso *et al.* (*Physical forces shape group identity of swimming Pseudomonas putida cells*)

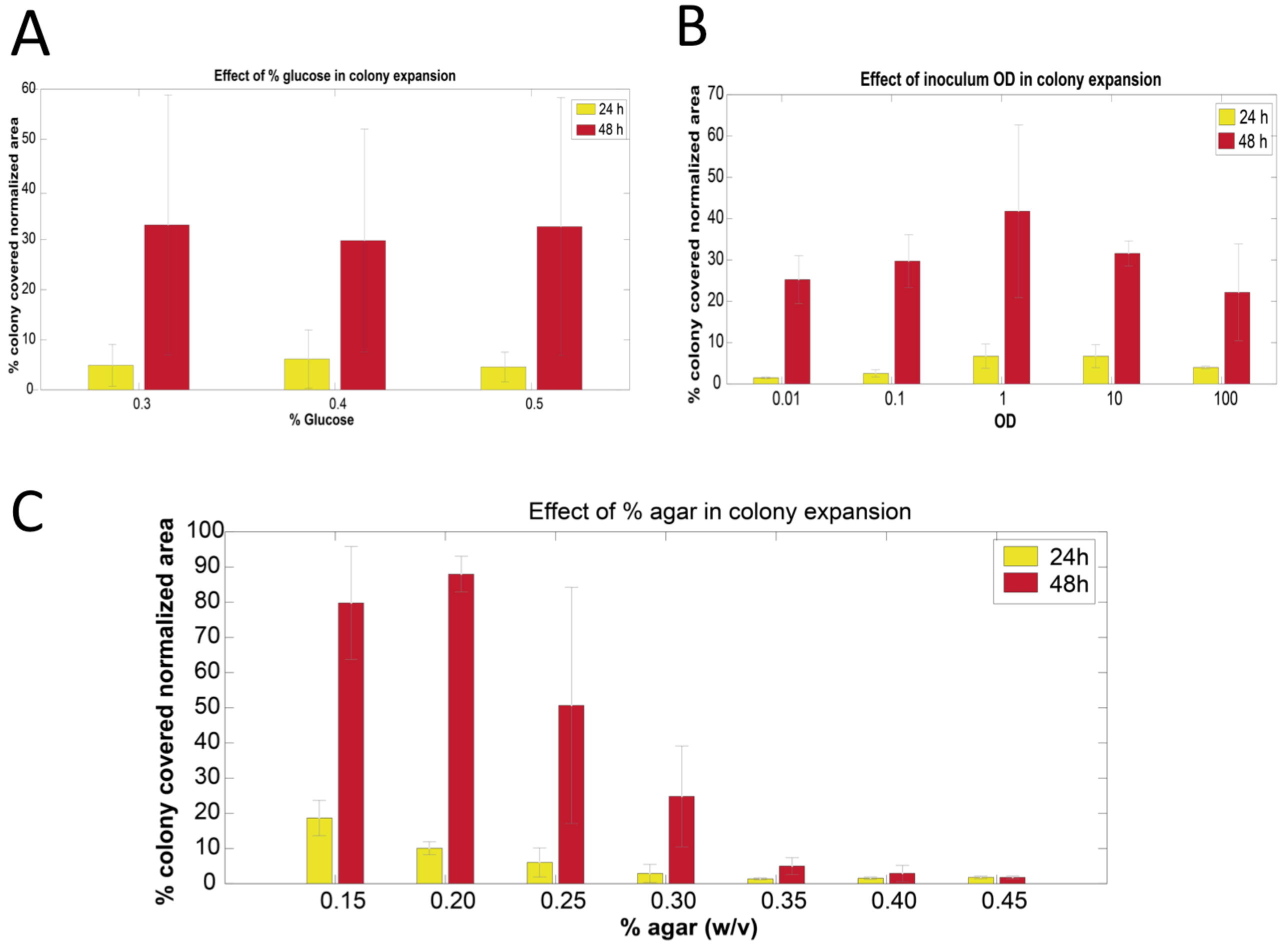

Quantification of swimming patterns of *P. putida* KT2440. Images obtained in the different experiments displayed in Figure 4 of the main article were quantified by applying the image treatment protocol described in Material and Methods. The effect of % of glucose (A), optical density of the initial inoculum (B) and % of agar in the plates (C) are displayed for the whole set of experiments. For each case, a minimum number of three biological repetitions with two technical samples were quantified. Data show mean values and standard deviations for the areas observed to be covered by the colonies (normalized with respect the total plate surface) at two different times: 24 h (yellow) and 48 h (red) as indicated.
